# Correlates of weight gain during long-term risperidone treatment in children and adolescents

**DOI:** 10.1186/1753-2000-6-21

**Published:** 2012-05-29

**Authors:** Chadi Albert Calarge, Ginger Nicol, Diqiong Xie, Bridget Zimmerman

**Affiliations:** 1 Departments of Psychiatry and Pediatrics, The University of Iowa Carver College of Medicine, 500 Newton Road, Iowa City, IA 52242, USA; 2 Department of Psychiatry, Washington University School of Medicine, 660 South Euclid Ave, Campus Box 8134, St. Louis, MO 63110, USA; 3 Departments of Psychiatry, The University of Iowa Carver College of Medicine, 500 Newton Road, Iowa City, IA 52242, USA; 4 Biostatistics Consultation Center, The University of Iowa College of Public Health, 200 Newton Road, Iowa City, IA 52242, USA

**Keywords:** Child, Adolescent, Weight gain, Obesity, Antipsychotics, Risperidone, Predictors

## Abstract

**Background:**

Most clinical trials of antipsychotics in children are brief, failing to address their long-term safety, particularly when taken concurrently with other psychotropics. This hypothesis-generating analysis evaluates potential correlates of weight gain in children receiving extended risperidone treatment.

**Methods:**

Medically healthy 7–17 year-old patients treated with risperidone for six months or more were enrolled. Anthropometric measurements were conducted. Developmental and medication history was obtained from the medical record. Information related to birth weight, dietary intake, physical activity, and parental weight was collected. Mixed regression analyses explored the contribution of various demographic and clinical factors to age- and sex-adjusted weight and body mass index (BMI) z scores over the treatment period.

**Results:**

The sample consisted of 110 patients (89% males) with a mean age of 11.8 years (sd = 2.9) upon enrollment. The majority had an externalizing disorder and received 0.03 mg/kg/day (sd = 0.02) of risperidone, for 2.5 years (sd = 1.7), to primarily target irritability and aggression (81%). Polypharmacy was common with 71% receiving psychostimulants, 50% selective serotonin reuptake inhibitors (SSRIs), and 32% α_2_-agonists. Weight and BMI z score were positively correlated with baseline weight at the start of risperidone, treatment duration, and the weight-adjusted dose of risperidone but inversely associated with the weight-adjusted dose of psychostimulants and the concurrent use of SSRIs and α_2_-agonists. The effect of risperidone dose appeared to attenuate as treatment extended while that of psychostimulants became more significant. The rate of change in weight (or BMI) z score prior to and within the first 12 weeks of risperidone treatment did not independently predict future changes neither did birth weight, postnatal growth, dietary intake, physical activity, or parental weight.

**Conclusions:**

This comprehensive analysis exploring correlates of long-term weight (or BMI) change in risperidone-treated youths revealed that pharmacotherapy exerts significant but complex effects.

**Trial Registration:**

Not applicable.

## Background

The use of atypical antipsychotics (AAPs) in children and adolescents has risen sharply over the last decade [1,2]. Several AAPs have received FDA approval for treatment of pediatric psychiatric conditions [3-6]. Moreover, AAPs in general, and risperidone in particular, significantly reduce irritability and aggression, which contributes to expanding their use to the more commonly-occurring disruptive behavior disorders [7,8]. Thus, given that further rise in the prescribing of AAPs to youths is anticipated, optimizing safety and tolerability is imperative. This is especially true since most pediatric psychiatric disorders targeted with AAPs are chronic, necessitating extended treatment [9,10].

One particularly concerning AAP-related adverse event is developmentally-inappropriate weight gain [11,12]. Obesity is an increasingly prevalent cardiometabolic risk factor leading to insulin resistance, dyslipidemia, and hypertension, among other sequelae [13]. Childhood obesity, specifically, is worrisome as it is strongly associated with adverse cardiometabolic conditions in adulthood [14]. Thus, AAP-induced weight gain in this vulnerable population has raised considerable concerns among clinicians and the public alike [15,16].

In fact, substantial evidence has linked the use of most AAPs to weight gain, with this outcome being particularly prominent in youths [12,17]. Still, significant inter-individual variability exists in the susceptibility to gain weight during AAP treatment, both in adult and pediatric samples [12,17]. Thus, identifying factors associated with weight gain during AAP treatment can help clinicians better estimate the risk/benefit ratio during the decision-making process.

To that end, research investigating childhood predictors of adult obesity can be informative. Specifically, such characteristics as birth weight [18], postnatal weight gain [19], physical activity [20], and dietary intake [20] have all been implicated in obesity risk. However, their contribution to the propensity to gain weight from AAPs has not been fully explored. Moreover, while a few studies have linked weight at the start of AAP treatment [11,21,22], AAP dose [12], the rate of weight gain during the first few weeks of AAP treatment [22,23], and polypharmacy [24] to AAP-induced weight gain in relatively short-term pediatric studies, these factors have not been thoroughly researched during long-term AAP treatment.

Thus, the aim of this hypothesis-generating analysis was to investigate whether the various demographic and clinical factors reviewed above affect weight gain during long-term risperidone treatment. Risperidone remains the best-studied and most widely used AAP in youth [1,5-7]. We hypothesized that low birth weight, rapid postnatal growth, and higher doses of risperidone will be associated with excessive weight gain. Conversely, high physical activity, a healthy diet, and co-treatment with psychostimulants will be associated with more age-appropriate weight gain.

## Methods

### Participants

This study has been previously described [11,25]. Briefly, 7 to 17 year-old patients treated with risperidone for six months or more were enrolled, irrespective of their primary psychiatric diagnosis or indication for risperidone. Concurrent treatment with additional psychotropics at enrollment, but not with other antipsychotics, was allowed. Subjects with neurological or medical conditions that could confound the metabolic assessments were excluded as were pregnant females and those receiving hormonal contraception. The primary aim of the study was to investigate the metabolic, hormonal, and skeletal adverse events of risperidone during long-term use.

### Procedures

This study was approved by the local Institutional Review Board. Written assent was obtained from children ≤ 14 years old and consent from adolescents and parents or guardians.

We recorded, from the medical record, the start and stop dates of each psychotropic, changes in the dosage and formulation, and the indication for risperidone. All dosages of psychostimulants were expressed in methylphenidate equivalents for amphetamines (x 2) [11]. We defined an SSRI unit as being equivalent to a daily dose of 10 mg of escitalopram, 20 mg of citalopram, fluoxetine, or paroxetine, and 50 mg of sertraline or fluvoxamine [25].

Upon enrollment, height was measured to the nearest 0.1 cm using a stadiometer (Holtain Ltd., UK) and weight was recorded to the nearest 0.1 kg using a digital scale (Scaletronix, Wheaton, IL), while wearing indoor clothes without shoes. In addition, we extracted, from the medical record, all height and weight measurements. When both were available, body mass index (BMI, kg/m^2^) was computed. However, since clinicians are more likely to measure weight than height, the database contained more than twice as many measurements for weight as for height or BMI. Importantly, measurements collected during clinic visits falling within a month of the research visits were highly correlated with the research measurements [mean interval = 16 days (sd = 9) for height (n = 69) and 17 days (sd = 9) for weight (n = 97)]. The intra-class correlations were all above 0.97 (95% confidence intervals [CI] ranging between 0.96 - 0.99) for unadjusted height and weight as well as for their sex- and age-adjusted z scores.

A best-estimate diagnosis, following the Diagnostic and Statistical Manual of Mental Disorders (DSM-IV-TR) [26], was generated based on a review of the psychiatric record often supplemented by a brief clinical interview, a standardized interview of the parent using the NIMH Diagnostic Interview Schedule for Children (DISC-IV; N = 95, 58%) [27], and the Child Behavior Checklist (N = 120, 74%) [28].

At enrollment, the parent completed a questionnaire regarding the pregnancy, the child’s birth weight, and current parental height and weight. The intraclass correlation coefficient between parental report and birth record was 0.91 for birth weight (95% CI = 0.86-0.95, n = 62) and 0.86 for duration of gestation (95% CI = 0.78-0.92, n = 57). Thus, parental report was used when the actual birth record could not be obtained. In addition, three groups were defined based on a change of 0.67 z score point in weight between birth and 2 years of life [19]. This magnitude of change has been recommended to select children with significant postnatal growth and corresponds to the width of each percentile band (e.g., 3, 10, 25, etc.) on standard growth charts [19].

The participants completed the 2004 Block Kids Food Frequency Questionnaire, with assistance from the parent when necessary [29]. This was reviewed by a research dietician to confirm completeness and accuracy. This questionnaire estimates the daily caloric intake and diet composition during the week prior to enrollment.

We also asked the subjects, with parental assistance when necessary, to list the five physical activities they participate in the most and to estimate the number of days per week that they engage in each one. This allowed computing the average number of days per week that the child engages in at least one of these five activities prior to enrollment. Furthermore, they reported the duration of their sleep and screen time (i.e., time spent watching television or playing video games and gameboy). Finally, using a five-point Likert scale, the parent compared their child’s activity level to same-age peers [11].

### Data analysis

This hypothesis-generating analysis aimed to identify which demographic and clinical variables were associated with weight gain during extended treatment with risperidone. However, in order to account for the natural growth observed in children monitored over several years, weight and BMI measurements were converted into age- and sex-adjusted z scores using the 2000 Center for Disease Control normative data [30]. A z score refers to the number of standard deviations above or below the mean, in a particular population. Similarly, a z score was generated for birth weight, adjusting for gender and gestational age [31].

In order to model weight (or BMI) z score change after risperidone was started, a hierarchical linear mixed model analysis was used to fit the trajectory of sex-age-adjusted weight (or BMI) z score over time by estimating a mean curve for weight (or BMI) z score over time for the overall group from the individual curve of each child. Thus, the model included both a fixed and random effect for intercept and slope (i.e., time), to represent the mean curve and the random variation of each child’s curve from the mean curve, respectively. The selected covariates of baseline weight (or BMI) z score (i.e., one obtained on or within a month before starting risperidone), weight-adjusted (mg/kg) daily dose of risperidone, age when risperidone was started, and weight-adjusted (mg/kg) daily dose of psychostimulants were included in this “primary” model since they have either been shown to affect AAP-induced weight (or BMI) gain or because there was a theoretical reason to believe so [11]. Interactive effects between time and other variables were tested and the significant ones were kept in the model. Because we anticipated that weight gain will plateau at some point after risperidone is started, the log transform of time was used in the model. Since BMI normally declines before it “rebounds” between 2.0 and 5.5 years of age, those measurements recorded before age 5.5 were excluded from the BMI analyses [30]. Finally, since the participants (7%) could have received antipsychotics other than risperidone prior to, but not at, enrollment, observations obtained after initiation of AAPs other than risperidone were excluded from the analysis.

Additional variables were subsequently added to the “primary” model to explore their independent contribution to risperidone-induced weight (or BMI) gain. When several related variables were tested (e.g., diet or physical activity), a backward model selection was applied to remove non-significant (α >5%) effects from the mixed regression model.

We also aimed to evaluate the association between change in weight prior to risperidone treatment with the change afterwards. For instance, we sought to explore whether children who might have lost weight before risperidone was started, presumably due to psychostimulants [32], exhibited accelerated weight gain with risperidone. Thus, we initially regressed weight (or BMI) z scores, obtained before starting risperidone, on time. This analytical method was used because, in this observational study, weight (or BMI) measurements were collected at the discretion of the clinicians, at varying time points during the course of treatment. The slope estimate of change in weight (or BMI) z score over time was standardized by dividing it by its own standard deviation in order to adjust for the variability in the number of observations used to estimate the slope for each subject. The standardized slope from this linear regression model was, subsequently, added to the “primary” linear mixed model as an additional predictor. A similar approach was used to model weight change during the first three months of risperidone treatment in order to explore whether it was associated with further weight gain.

All the statistical tests performed were two-tailed, using SAS version 9.2 for Windows (SAS Institute Inc., Cary, NC). Since this was a hypothesis-generating analysis, we did not correct for the number of tests conducted.

## Results

### Subject characteristics

A total of 163 participants were recruited into the study of whom 110 (67%) had the necessary data to contribute to testing the “primary” model. The single most common reason for exclusion from the analysis was the absence of a baseline weight in 26% of the sample. The demographic and clinical characteristics of those contributing to this analysis are reported in Table 1. Across all the demographic and clinical variables listed in Table 1 through 3, only weight z score (but not BMI z score) was different between those included and excluded from the analysis.

**Table 1:** Demographic and Anthropometric Characteristics of the Sample


**Characteristic**	
Age, years	11.8 (±2.9)
Males, n (%)	98 (89%)
Tanner Stage: I, II, III, IV, V (%)	36/14/18/22/10
Race, n (%)	
White	87 (79)
African American	17 (15)
Hispanic	5 (5)
Other	1 (1)
**Anthropometric/Metabolic Data**	
Birth Weight z Score^a^ (n=98)	-0.29 (±0.99)
Baseline Weight z Score^b^	0.17 (±1.02)
Baseline BMI z Score^b^ (n=107)	0.18 (±1.05)
Enrollment Weight, Kg	49.1 (±18.3)
Enrollment Weight z Score	0.72 (±1.03)
Enrollment Weight, Kg/m^2^	20.9 (±4.6)
Enrollment BMI z Score	0.73 (±1.02)

^a^ This was adjusted for sex and gestational age [31].

_b_ Baseline sex- and age-adjusted weight or body mass index (BMI) z scores included those available upon or within the month prior to starting risperidone.

The mean age at enrollment of the participants was 11.8 years (sd = 2.9) and the mean duration of treatment with risperidone was 2.5 years (sd = 1.7). The majority (81%, n = 89) received risperidone to target irritability and aggression, after having failed to respond to other psychotropics over 2.3 years (sd = 2.3).

As shown in Table 1, attention deficit hyperactivity disorder (ADHD) and disruptive behavior disorders were most common. As is typical for such a clinical sample, comorbidity was prevalent, with a median number of diagnoses of 2 (Interquartile range [IQR] = 1). Moreover, polypharmacy was the rule with only 4% (n = 4) of the sample taking risperidone alone. In fact, the median number of psychotropics, taken in addition to risperidone, was 3 (IQR = 1), with psychostimulants, selective serotonin reuptake inhibitors (SSRIs), and α2-agonists (i.e., clonidine or guanfacine) being the most co-prescribed (Table 2). Other psychotropics received upon enrollment by a minority of participants include trazodone (n = 5), imipramine (n = 4), clomipramine (n = 3), valproate (n = 2), bupropion (n = 2), gabapentin (n = 2), lithium (n = 1), oxcarbazepine (n = 1), topiramate (n = 1), lamotrigine (n = 1), levetiracetam (n = 1), buspirone (n = 1), and venlafaxine (n = 1).

As previously reported [11], weight and BMI z scores significantly increased, after risperidone was started, by nearly 0.5 z score point (p<.0001) (Table 1).

**Table 2:** Clinical Characteristics of the Sample


**Characteristic**	
**Psychiatric Diagnoses, n (%)**	
Attention Deficit Hyperactivity Disorder	96 (87)
Disruptive Behavior Disorder	100 (91)
Anxiety Disorder	36 (33)
Tic Disorder	24 (22)
Depressive Disorder	12 (11)
Pervasive Developmental Disorder	14 (13)
Psychotic Disorder	1 (1)
**Pharmacotherapy**	
Risperidone Dose, mg/d	1.4 (±1.1)
Risperidone Dose, mg/kg/d	0.03 (±0.02)
Age at Start of Risperidone, yrs	9.1 (±2.8)
Treatment Duration, yrs	2.5 (±1.7)
Psychostimulants, n (%)	78 (71)
Psychostimulants Dose, mg/d ^a^	59.2 (±28.6)
Psychostimulants Dose, mg/kg/d ^a^	1.35 (±0.70)
Psychostimulants Treatment Duration, yrs	4.8 (±2.7)
SSRIs, n (%)	55 (50)
Fluoxetine	21 (19)
Sertraline	16 (15)
Citalopram/Escitalopram	18 (16)
SSRI Dose, unit per day ^**b**^	1.30 (±0.83)
SSRI Treatment Duration, yrs	2.6 (±2.1)
α_2_-agonists, n (%)	35 (32)

^a^ All dosages of psychostimulants were expressed in methylphenidate equivalents for amphetamines (x 2) [33].

^b^ One SSRI unit is equivalent to a daily dose of 10 mg of escitalopram, 20 mg of fluoxetine, citalopram, or paroxetine, and 50 mg of sertraline or fluvoxamine [25].

### Weight gain during risperidone treatment; “primary” model

Over a period extending between 15.6 years before and 8.4 years after risperidone was started, a mean of 15.1 (sd = 12.2) weight measurements were available per month for the sample. More than twice as many measurements (mean = 31.7, sd = 10.9) per month were available for the period extending between 2.5 years before and 2.5 years after risperidone was started.

Using mixed regression analysis to test the “primary” model, baseline weight z score (β = 1.13, p < .0001), time on risperidone (β = 0.16, p < .0001), and the weight-adjusted dose of risperidone (β = 2.6, p < .0001) were all positively associated with weight z score. In addition, the interaction effect of time and baseline weight z score was significant (β = −0.05, p <; .005) as well as that of time and the weight-adjusted dose of psychostimulants (β = −0.04, p <; .0001). This suggests that, over time, the association between baseline weight z score and subsequent measurements attenuates while that of psychostimulants becomes more prominent. There was no significant effect of age when risperidone was started (p > .5).

Similarly, baseline BMI z score (β = 1.05, p <; .0001), time on risperidone (β = 0.16, p <; .0001), and the weight-adjusted dose of risperidone (β = 14.0, p <; .0001) were positively associated with BMI z score with the weight-adjusted dose of psychostimulants being negatively so (β = −0.19, p <; .0001). Again, the effect of baseline BMI z score wanes over time (interaction effect β = −0.05, p <; .02). In addition, the interaction effect of time and the dose of risperidone was significant (interaction effect β = −1.96, p <; .001), also suggesting that the association between the dose of risperidone and BMI z score declines over the course of treatment.

### Effect of concomitant psychotropics

Besides psychostimulants, SSRIs and α_2_-agonists were the most commonly co-prescribed psychotropics in this sample (Table 2). Thus, we investigated their potential effect on risperidone-induced weight gain. The number of SSRI units was negatively associated with weight z score (β = −0.05, p = .0005) while both SSRIs and α_2_-agonists were negatively associated with BMI z score (β = −0.06, p <; .04 and β = −0.14, p = .0006, respectively), after accounting for the other “primary” covariates.

### Effect of birth weight and growth rate in infancy

Birth weight z score, adjusted for gender and gestational age, was significantly correlated with weight z score at the onset of psychotropic treatment (Spearman’s r = 0.29, p = .0007) but not with BMI z score (Spearman’s r = 0.13, p > .1). However, it did not significantly contribute to the mixed regression model of either weight or BMI z score (both p values > .1), after accounting for the other “primary” covariates.

Between birth and 2 years of life, weight z score increased by an average of 0.6 (sd = 1.3). Of the 70 participants who had this information available, 14% (n = 10) showed a reduction of their weight z score by more than 0.67 point, 50% showed no significant change, and 36% (n = 25) showed an increase by more than 0.67 point (i.e., catch-up group). This postnatal change in weight z score did not significantly influence weight or BMI z score during risperidone treatment, after accounting for the other “primary” covariates (all p values > .1).

### Effect of pre-risperidone change in weight or BMI z scores

Next, we explored whether the change in weight during the 18 months prior to risperidone initiation improved model prediction of weight gain in risperidone-treated children. As described above, we estimated the slope of change in weight z score for each individual and standardized it before entering it in the mixed regression model as an additional covariate. Data were available to compute this slope in 96 participants, of whom 33 had a negative slope (i.e., they lost weight during that period), 45 had a null slope, and 18 had a positive one. After accounting for the “primary” covariates, the slope of weight z score did not significantly contribute to the model. Similar results were found for BMI z score.

### Effect of acute post-risperidone change in weight or BMI z scores

We, then, explored whether the change in weight during the first three months after risperidone was started was associated with further weight z score changes. Again, we estimated the slope of change in weight z score for each individual and standardized it before entering it in the mixed regression model as an additional covariate. Data were available to compute this slope in 40 participants for the weight and in 17 for the BMI z score analyses. After accounting for the “primary” covariates, the slope of change in weight z score during the first 3 months of treatment did not significantly contribute to the model. However, there was a suggestion of a positive association between the change in BMI z score during the first 3 months of risperidone treatment and further increase in BMI z score (β = 0.10, p <; .1), after taking into account the other “primary” covariates.

### Effect of dietary intake, physical activity, and parental BMI

Table 3 lists the mean values of the different variables related to dietary intake and physical activity. After controlling for the other covariates in the “primary” model, neither dietary intake, nor physical activity, maternal BMI, or paternal BMI obtained at enrollment significantly contributed to the change in weight or BMI z scores.

**Table 3:** Dietary Intake and Physical Activity in the Sample


**Characteristic**	
**Dietary Intake**	
Total Calories, kcal/d	1926 (±772)
Protein Content, %	14.1 (±2.3)
Fat Content, %	32.1 (±4.4)
Carbohydrates Content, %	55.3 (±6.2)
**Physical Activity**	
Sleep, hrs/day	8.6 (±3.1)
Screen Time, hrs/day ^a^	2.9 (±2.1)
Days of Activity per Week	3.5 (±1.2)
Parent-rated Activity ^b^	2.4 (±1.1)

^a^ Screen time included the time spent watching television, playing video games or on the computer, etc.

^b^ Parent-rated activity was based on a single-item question asking the parent to compare the child’s level of activity to his peers on a 5-point Likert scale with 2 being equivalent to peers.

## Discussion

To our knowledge, this is the first pediatric study, spanning several years before and after the onset of risperidone treatment, to identify several independent correlates of change in weight (BMI). The possible confounding effect of other AAPs was removed by restricting this analysis to a single agent, risperidone, which is the most commonly-used and best investigated antipsychotic in youths [1,5-7]. In addition, this sample included a heterogeneous clinical group, representative of the general population treated with AAPs. While baseline weight (or BMI) z score and the daily dose of risperidone were associated with a higher weight (or BMI) z score during risperidone treatment, these effects subsided over time. On the other hand, psychostimulants, SSRIs, and α_2_-agonists were associated with lower weight (BMI).

### Dose-dependent weight gain with risperidone

Studies in adults suggest that for some, but perhaps not all, AAPs, weight gain and cardiometabolic abnormalities are dose-dependent [34]. In children and adolescents, short-term, randomized clinical trials and observational studies have found weight gain to be dose-dependent for risperidone and aripiprazole but not quetiapine [6,12,35]. The findings with risperidone are inconsistent, however, perhaps because, in the one clinical trial in bipolar disorder that failed to find a dose effect, the treatment groups were not matched on baseline weight [36]. Regardless, because of the short-term duration of these trials, it is difficult to make predictions about weight changes over longer periods of exposure. Moreover, most pediatric clinical trials conducted to date have not enrolled participants based on weight at the onset of treatment or AAP-naïve status, two related characteristics associated with AAP-induced weight gain [11,12,22,37,38].

Another critical factor influencing a potential link between AAP dose and weight gain is medication adherence. Adherence is often suboptimal during extended treatment of any kind, but perhaps particularly so in the population of children receiving AAPs. Furthermore, treatment adherence is often challenging to capture accurately in naturalistic long-term studies such as the one reported here. This is particularly important since several antipsychotic-related adverse events that could potentially reduce adherence, such as sedation or hyperprolactinemia, are dose-dependent [36,39,40]. Finally, adjusting the dose for weight, as we have done, or measuring the medication serum concentration could increase the sensitivity of dose as a predictor of weight gain.

The factors mentioned above might explain some of the inconsistency in the literature regarding the dose effect of AAPs on weight gain. Moreover, we found that the dose of risperidone is associated with an increase in BMI z score but this effect wanes over time. This too could explain why only some studies have found a dose effect. Since weight gain eventually plateaus, following an interval whose duration is related to several factors including baseline weight and status of prior treatment with AAPs, it is likely that dose exerts a significant effect during the initial phase of treatment but this gradually wanes as weight gain nears its plateau. In other words, our findings suggest that youth receiving a lower dose of risperidone will gain weight at a slower rate. However, during extended treatment, the eventual weight accrued might be similar to when higher doses are used, since each individual will eventually reach his/her new homeostatic set point, as modified by AAP treatment. The metabolic consequences of these differing trajectories have been reported elsewhere [41].

### Contribution of concurrent psychotropic medications

**Figure 1:**
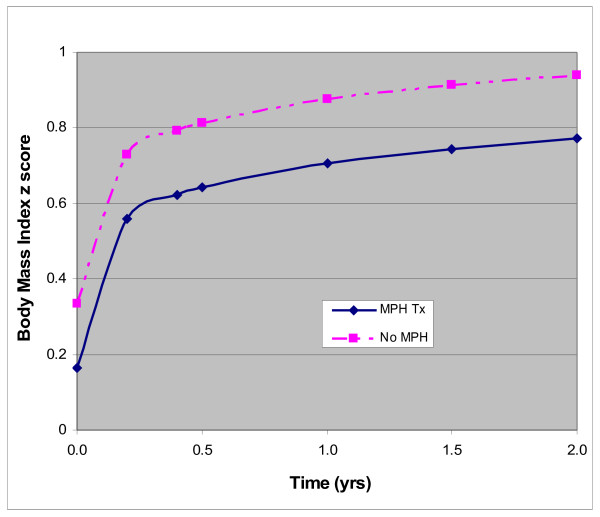
**Trajectory of weight gain with and without treatment with psychostimulants.** Estimated trajectory of BMI z score change over a two-year period in a hypothetical participant on risperidone monotherapy and one on a combination of risperidone and a psychostimulant. These trajectories were modeled for a prototypical participant using the sample mean for baseline BMI z score (0.13), age at the onset of risperidone treatment (9.3 years), daily weight-adjusted dose of risperidone (0.03 mg/kg/d) and methylphenidate (0.9 mg/kg/d).

Psychostimulants are known to suppress appetite and growth [32]; thus, investigators have explored whether these medications protect against AAP-related weight gain. Two large, short-term, studies failed to find a “protective” effect of concurrent psychostimulant treatment [12,42]. A third, albeit smaller, study showed a similar lack of effect [43]. However, such studies usually require maintaining the dose of psychostimulants stable for a period of time prior to and during AAP treatment. Unlike in our prior publication where concomitant exposure to psychostimulants was simply averaged across the risperidone-treatment period [11], our current, more precise, analysis revealed that a weight-adjusted dose of concomitant psychostimulant therapy was associated with less weight gain and that this effect seemed to persist over time. Figure 1 illustrates the trajectory of BMI z score of two, hypothetical, otherwise identical risperidone-treated children except that only one of them is receiving concurrent treatment with a psychostimulant.

In adults, acute SSRI monotherapy tends to either be weight neutral or to reduce weight. However, an increase in weight has been noted over longer periods of treatment [44]. This effect varies across the different SSRIs, with fluoxetine being least associated with weight gain and paroxetine most [44]. While short-term studies have described similar findings in children [45,46], to our knowledge, only one intermediate-term placebo controlled pediatric trial of an SSRI reported weight changes. In that multiphase study, fluoxetine led to a reduction in weight during the first 19 weeks [47]. This appears to have partially recovered by week 51, though the study suffered from a significant attrition [See authors reply in ref [48]]. Fluoxetine was the most commonly used SSRI in our sample and no one received paroxetine (Table 2), perhaps explaining why SSRI treatment was associated with less weight gain in our sample. The limited number of participants receiving various SSRIs, however, does not allow us to explore their differential effect. Of note is that fluoxetine is a potent inhibitor of the CYP2D6 enzyme which is the predominant pathway for risperidone metabolism. However, the active moiety serum concentration (i.e., combined risperidone and 9-hydroxyrisperidone concentration) is at most minimally increased [49,50]. This could, theoretically, accentuate weight gain from risperidone, although the effect is likely negligible. One significant challenge is how to validly capture effective SSRI exposure across varying formulations, developmental stages, and levels of medication adherence. We used the formula described above because of the ease of its clinical use. However, serotonin transporter occupancy might be more accurate had this information been available for children as for adults [51,52].

Alpha2-agonists can cause anorexia acutely but have also been reported to cause weight gain [53]. To our knowledge, the long-term effect of α2-agonists on weight in youth has not been investigated in controlled studies. Therefore, our finding of an inverse association between the use of α2-agonists and BMI z score awaits replication but highlights the complex potential outcomes when multiple psychotropics are combined.

One note of caution is that the negative associations between SSRIs and α2-agonists and weight do not imply that these medications are “protective” against risperidone-induced weight gain. Rather, they merely suggest that risperidone-treated children who are concurrently taking SSRIs or α2-agonists tend to have lower weight/BMI. Therefore, our findings should be interpreted cautiously and not considered to justify polypharmacy.

### Contribution of birth weight, caloric intake, physical activity, and parental weight

Several studies have linked birth weight to weight later in life [18]. Similarly, an association between rapid growth in infancy or childhood and excessive weight and insulin resistance later on has also been documented [19]. These findings are believed to support the “thrifty phenotype” hypothesis purporting that nutritionally-depriving conditions program the body to optimize energy conservation [54]. When followed by free access to energy-rich food, this “homeostatic reprogramming” could lead to excessive fat accumulation and the development of obesity and insulin resistance [54]. We hypothesized that the “thrifty phenotype” would accentuate the increased energy intake or conservation associated with risperidone treatment, thus leading to more weight gain. However, neither birth weight nor postnatal growth appeared to significantly contribute to risperidone-related weight gain in our sample, after the other significant factors were taken into account.

The contribution of high-calorie diets and a sedentary lifestyle to the obesity epidemic in the world is well documented [20]. Moreover, interventions that modify either one of these factors have resulted in weight loss [20]. More specifically, AAPs induce weight gain primarily by increasing appetite and caloric intake [55-57], though they could reduce physical activity due to sedation. We failed to find such an association, perhaps because we collected these data long after risperidone had been initiated and weight gain stabilized. This might also explain why we did not find an association between parental BMI and the children’s propensity to gain weight.

### Contribution of weight trajectories before and after risperidone initiation

The discontinuation of psychostimulants is followed by accelerated growth [58]. Conversely, as noted earlier, prior treatment status is a strong correlate of AAP-induced weight gain. Nonetheless, we found no significant association between change in weight prior to the onset of risperidone and the change afterwards.

Weight gain during the first few weeks of olanzapine treatment is a strong predictor of future change in weight [22,59]. We found only a statistical trend suggesting that an increase in BMI z score during the first three months of treatment is associated with further weight gain during more extended treatment, after taking into effect the other “primary” covariates. However, the sample size in this analysis was too small and requires future replications.

The results reported here are subject to limitations. First, it would be ideal if a randomized trial involving various AAPs explored both efficacy and tolerability during extended exposure. However, such a study is challenging to conduct for ethical, budgetary, and practical reasons. Alternatively, studies such as this one can help fill the gap by providing sorely-needed information about the long-term safety of AAPs using a more pragmatic design. Nonetheless, this remains a retrospective study with missing data, particularly regarding patients who discontinue risperidone early on and, as a result, are not enrolled. It is somewhat reassuring that the amplitude of weight gain we found is comparable to that reported in shorter-term studies [12]. In addition, nearly a third of our participants (53/163) were excluded from the analysis due to missing data. They differed from the included participants on enrollment weight, but not BMI, z score. Thus, this finding could be due to a type I error since it was the only variable that was significantly different between the two groups across 39 tested. Secondly, the specific aim of the parent study was to investigate the metabolic and skeletal effects of risperidone specifically in youths who have been receiving it over extended periods of time. It is in these children that one would be most concerned about long-term AAP safety, making the results both relevant to common clinical practice and generalizable to the broad population of children treated with AAPs. Third, except for those obtained upon study enrollment, all anthropometric measurements were not necessarily collected following standard procedures. However, like others [60], we found weight and height measurements extracted from the medical record to be highly correlated with the corresponding research measurements, obtained within a month. Further, we contend that it is the trajectory of change in weight (or BMI) during the course of treatment, as opposed to single measurements, that is of most clinical relevance. Modeling the weight (BMI) trajectory requires serial measurements, making it less susceptible to measurement errors. Fourth, we used the prescribed dose of risperidone as a predictor rather than risperidone serum concentration. This is particularly important as the serum concentration, had it been obtained repeatedly over the treatment course, would have better captured medication adherence. As discussed earlier, long-term adherence is challenging to estimate accurately and tends to decrease over time. In fact, this phenomenon could potentially explain the apparent attenuation of the effect of risperidone dose on weight that we found. Fifth, while comorbidity was common, the prevalence of various disorders was somewhat bimodal with ADHD and disruptive behavior disorders being almost universal with the other disorders afflicting a relative minority. This precluded our ability to explore whether the underlying psychiatric disorders moderated risperidone-related weight gain. Short-term randomized pediatric trials in disruptive behavior, bipolar, psychotic, tic, and pervasive developmental disorders have all revealed a somewhat comparable weight gain with AAPs [22,36,61-64]. However, none of these studies directly compared weight gain across different disorders, leaving unanswered the question of whether psychopathology moderates one’s propensity to gain weight during AAP treatment. Also, with the heritability of AAP-induced weight gain estimated at 60 to 80% [65], it is likely that genetic factors contribute to this adverse event as we and others have shown [66,67]. These were not considered in the present analysis but should be in future ones. Finally, it is important to replicate our findings in a larger sample where females and individuals from a diverse racial/ethnic background are better represented.

## Conclusions

In sum, we investigated the role of several demographic and clinical factors in weight gain during extended risperidone treatment in children. We found that baseline weight and the dose of risperidone were associated with weight gain but their effects subside as exposure continues. On the other hand, commonly co-prescribed medications, including psychostimulants, SSRIs, and α2-agonists, were associated with lower weight. Finally, additional putative risk factors, like birth weight, postnatal growth, physical activity, and dietary intake appear to play a limited role, if any.

While no single intervention will be sufficient to address a multi-factorial clinical problem like balancing the risk/benefit ratio of AAP treatment, our findings shed light on the complexity of how risperidone and commonly prescribed psychotropics influence weight gain and obesity risk during long-term treatment in youths. They specifically suggest that psychotropics play a potentially important role in altering the trajectory of weight gain during development. This effect might have implications in the subsequent development of cardiometabolic abnormalities and, therefore, offers an opportunity for preventive interventions.

## Competing interests

The authors report no competing interests.

Dr. Calarge has received research grant support from the National Institute of Mental Health, (NIMH), the National Alliance for Research in Schizophrenia and Depression (NARSAD), and the Children’s Miracle Network. Dr. Nicol has received research grant support from the National Institute of Mental Health, (NIMH), the National Alliance for Research in Schizophrenia and Depression (NARSAD), the Sydney R. Baer, Jr. Foundation, the Communities Helping Adolescent Depression and Suicide (CHADS) Coalition for Mental Health, the Dana Brown Charitable Trust Foundation and Pfizer Inc. She also receives royalties from Jones & Bartlett Learning for a pediatric metabolic monitoring form. Dr. Zimmerman and Dr. Xie have no conflict of interest to disclose.

## Authors’ contributions

All authors have given final approval of the submitted version of the manuscript. CC: Designed and conducted the study, contributed to data analysis/interpretation and manuscript preparation. BZ and DX: Conducted the data analysis and contributed to the interpretation of findings and to manuscript preparation. GN: Contributed to the interpretation of findings and to manuscript preparation.
